# Biclonal IgD and IgM Plasma Cell Myeloma: A Report of Two Cases and a Literature Review

**DOI:** 10.1155/2013/293150

**Published:** 2013-11-18

**Authors:** Zhongchuan W. Chen, Ioanna Kotsikogianni, Jay S. Raval, Christine G. Roth, Marian A. Rollins-Raval

**Affiliations:** ^1^Department of Laboratory Medicine and Pathobiology, University of Toronto, Toronto, ON, Canada M5S 1A1; ^2^Pathology Laboratory, Patras General Hospital “O Agios Andreas,” 26500 Patras, Greece; ^3^Department of Pathology, University of Pittsburgh, Pittsburgh, PA 15261, USA; ^4^The Institute for Transfusion Medicine, Pittsburgh, PA 15213, USA; ^5^Department of Pathology and Laboratory Medicine, University of North Carolina, Chapel Hill, NC 27599-7525, USA

## Abstract

Biclonal plasma cell myelomas producing two different isotypes of immunoglobulins are extremely rare entities; to date, the combination of IgD and IgM secretion by a biclonal plasma cell myeloma has not been reported. Bone marrow biopsy immunohistochemical studies in two cases revealed neoplastic plasma cells coexpressing IgD and IgM, but serum protein electrophoresis identified only the IgM monoclonal paraprotein in both cases. Biclonal plasma cell myelomas, while currently not well characterized in terms of their clinical behavior, should be distinguished from B-cell lymphoma with plasmacytic differentiation, given the different therapeutic implications. Both cases reported herein demonstrated chemotherapy-resistant clinical courses.

## 1. Introduction

Immunosecretory disorders are clonal proliferations of immunoglobulin-producing plasma cells or lymphocytes that secrete a single isotype or polypeptide subunit of immunoglobulin (Ig) usually detectable as a monoclonal protein peak (M-protein) on serum or urine protein electrophoresis studies. Most plasma cell myelomas (PCMs) result in a monoclonal gammopathy, with IgG M-protein produced in slightly more than 50% of cases and IgA in 20% of cases [1, 2]. Another 20% of cases produce only monoclonal light chains [1]. Fewer than 2% of cases produce monoclonal IgD, IgE, or IgM [3, 4]. 

Only rare PCMs result in biclonal gammopathy with the production of two different heavy chains and/or light chains. In a large review of 1027 PCM patients, only 2% had a biclonal gammopathy on protein electrophoresis studies [2]. However, the review did not specify which combinations of biclonal M-proteins were present. Other reports have described combinations of biclonal gammopathies, including IgD/IgG, IgG/IgM, IgA/IgG, and kappa/lambda light chain biclonal gammopathies [3, 5–9]. We report herein two cases of IgD/IgM biclonal PCM, a combination of heavy chain production that has not been previously described in the literature. 

## 2. Case Presentations

### 2.1. Case  1

**Figure 1:**
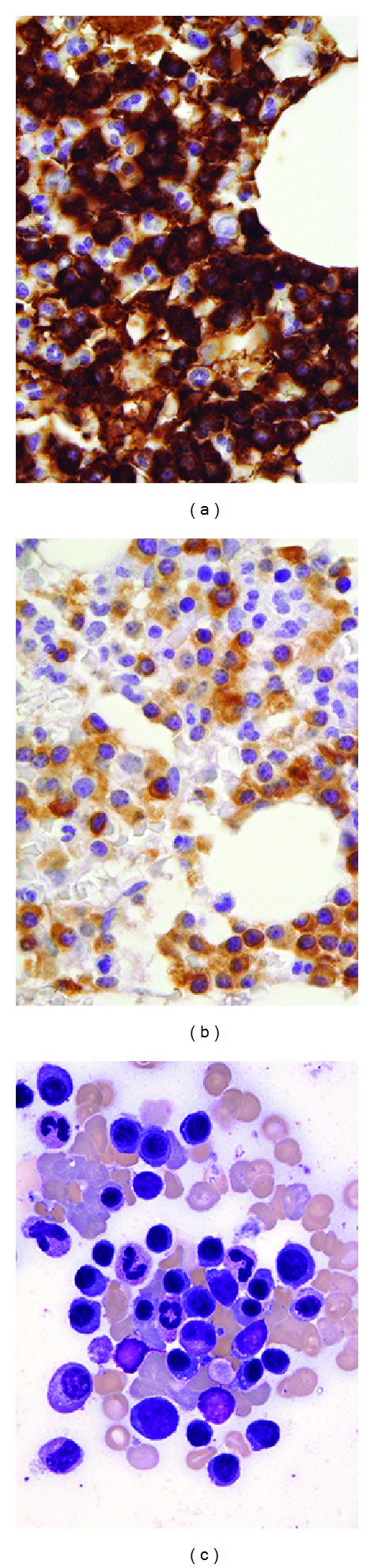
*Case 1.* (a) Biopsy from initial sample demonstrating that the majority of cells express IgM. (b) A subset of cells express IgD, (c) Aspirate smear of second pretreatment marrow evaluation. Immunohistochemistry, oil objective, original magnification ×500 ((a) and (b)); Wright-Giemsa, oil objective, original magnification ×1000 (c).

A 55-year-old male presented with anemia (hemoglobin 8.5 g/dL, reference range 14–17 g/dL). He had been on warfarin therapy following aortic valve replacement and mitral valve repair due to a recent episode of bacterial endocarditis. His medical history was also significant for diabetes mellitus, sarcoidosis, hypothyroidism, and hypertension. A bone marrow biopsy was performed as part of the anemia evaluation. The aspirate smears were suboptimal in preparation, but the bone marrow biopsy demonstrated normocellular marrow with a diffuse interstitial infiltrate of plasma cells comprising more than 30% of the marrow elements. The plasma cells were mildly atypical, with a rare Dutcher body identified. Flow cytometry performed on the aspirate specimen demonstrated that the CD138 positive plasma cells were CD56 positive and exhibited surface and cytoplasmic lambda light chain restriction. Flow cytometric studies did not identify an abnormal B-lymphoid population. Immunohistochemistry performed on paraffin embedded sections of the bone marrow biopsy revealed the neoplastic cells to be CD138 positive, CD20 negative, IgM heavy chain positive (Figure 1(a)) and lambda light chain restricted. Interestingly, a subset of these cells expressed IgD heavy chain (Figure 1(b)), and cyclin D1. None of the cells expressed IgA or IgG. Fluorescence in-situ hybridization (FISH) analysis for CCND1/IGH fusion, indicating a t(11;14), was negative in 99% of the cells using Vysis DNA probes (Abbott Molecular Inc., Des Plaines, IL, USA). Following the bone marrow biopsy, serum protein electrophoresis demonstrated a monoclonal peak in the beta region (1.4 g/dL) with immunofixation confirming an IgM-lambda monoclonal gammopathy. Immunofixation for IgD was not assessed. Biochemical analysis revealed a borderline low ionized calcium level (0.93 mmol/L, reference range 1.0–1.4 mmol/L), and normal blood urea nitrogen and creatinine levels. No lytic lesions were observed by radiographic imaging. At this point, the neoplasm was best considered asymptomatic (smoldering) myeloma, as the patient had more than 10% clonal plasma cells in the bone marrow, but no organ or tissue impairment was attributed to the neoplasm. Three months later, the patient underwent a second surveillance bone marrow biopsy. This time, the morphology of the neoplastic cells was evaluable on the marrow aspirate smear and was “lymphoplasmacytoid” (Figure 1(c)). The neoplastic cells accounted for 35% of the total cellularity based on the marrow aspirate smear differential. Immunohistochemistry was not performed on the bone marrow biopsy, but flow cytometric studies confirmed the persistent CD138 positive and CD56 positive lambda monoclonal plasma cell population that was negative for CD19 and CD20. Since his serum IgM level was elevated to 4660 mg/dL (reference range 40–230 mg/dL) with depression of IgA and IgG levels, he was started on dexamethasone, vincristine, and doxorubicin; however, this therapy did not decrease IgM levels and he was switched to a thalidomide/dexamethasone regimen. The dexamethasone was stopped eight months later due to uncontrollable hyperglycemia. He was continued on the thalidomide, and his IgM levels decreased to 2270 mg/dL and appeared stable. However, within three months, his IgM levels increased to 3420 mg/dL and thalidomide was discontinued. A third bone marrow biopsy at this time demonstrated persistent disease with neoplastic plasma cells accounting for 23% of the total cellularity based on the marrow aspirate differential. Serum protein electrophoresis continued to exhibit an IgM-lambda monoclonal protein (0.17 g/dL) as well as a second nonquantifiable free lambda light chain protein. At this time, now two years after his initial diagnosis, the patient underwent high dose melphalan autologous peripheral blood stem cell transplantation.

A fourth bone marrow biopsy performed one year later demonstrated disease progression with 50% neoplastic plasma cells based on manual marrow aspirate differential and 12% plasma cells in the peripheral blood. At this time, he was started on lenalidomide, but unfortunately the new therapy was not tolerated. A subsequent bone marrow biopsy three months later demonstrated continued progression with 82% plasma cells. At this point, there was also an evolution to plasma cell leukemia, evidenced by 28% circulating plasma cells. Within six months, he underwent a second autologous peripheral blood stem cell transplantation complicated by pancytopenia requiring growth factor support and red blood cell and platelet transfusions. The transplantation was ultimately unsuccessful and his plasma cell leukemia, despite reinitiation of lenalidomide and dexamethasone, persisted. His clinical course was complicated by lower extremity cellulitis, *Klebsiella pneumoniae* sepsis, and uncontrollable hematuria, and the patient died 4 years and 4 months after his initial diagnosis with persistent PCM. 

### 2.2. Case  2

A 71-year-old male presented with altered mental status and was initially diagnosed with streptococcal pneumonia, sepsis, and left lower extremity deep venous thrombosis. Peripheral blood examination revealed anemia (hemoglobin 7.8 g/dL, reference range 12.9–16.9 g/dL) with 9% plasma cells and significant rouleaux formation. Quantitative immunoglobulin determination showed a marked increase in IgM (7655 mg/dL, reference range 40–274 mg/dL). Serum protein electrophoresis with immunofixation revealed monoclonal IgM-kappa M-protein (5.34 g/dL). Urine protein electrophoresis demonstrated the presence of two monoclonal spikes in the gamma region (13.28 mg/dL and 104.69 mg/dL), and immunofixation performed on the urine demonstrated a kappa light chain not associated with IgA, IgG, or IgM heavy chains. CT scan of the abdomen and pelvis displayed neither lymphadenopathy nor hepatosplenomegaly, and a radiographic skeletal survey was negative for lytic lesions. Due to the patient's deteriorating clinical status, which included worsening altered mental status attributed to hyperviscosity (serum viscosity 8.8 cP, reference range 1.4–1.8 cP) from elevated IgM, a course of therapeutic plasma exchange (TPE) was initiated. Ten 1.5 volume TPE procedures with 5% albumin replacement were performed over a 20-day period to reduce IgM levels and improve cognitive impairment. Due to clinical suspicion for Waldenström macroglobulinemia, a bone marrow evaluation was performed.

**Figure 2:**
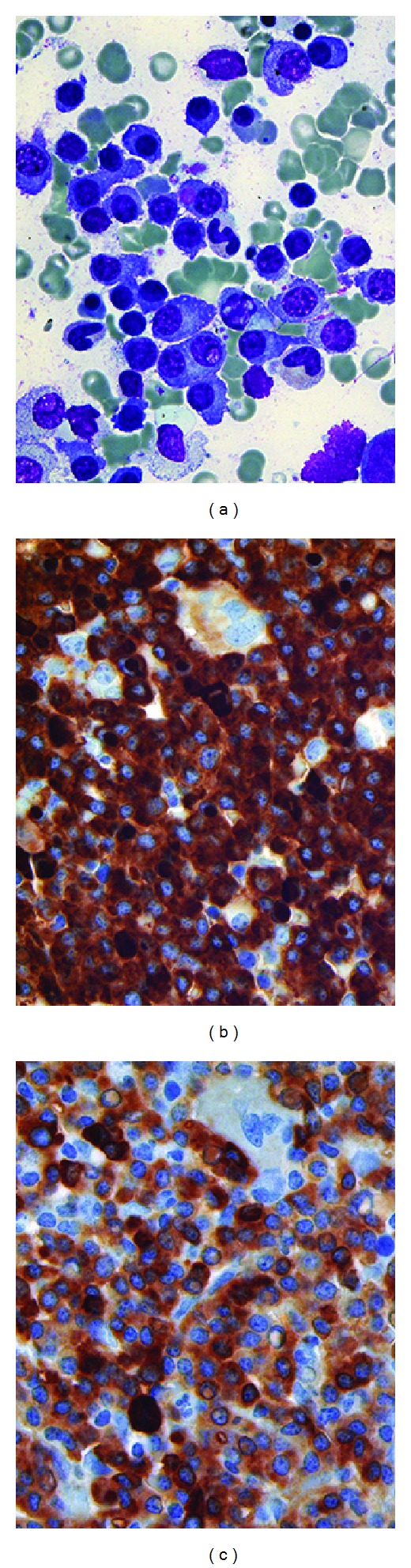
*Case 2.* (a) Bone marrow aspirate reveals large numbers of mature-appearing plasma cells with lymphoplasmacytoid morphology, some of which contain Dutcher bodies and cytoplasmic immunoglobulin inclusions. (b) Immunohistochemical studies on the trephine biopsy demonstrate that almost all cells express IgM, some with Dutcher bodies. (c) A subset of cells also express IgD but at a weaker intensity than those expressing IgM. Wright-Giemsa, original magnification ×1000 (a); IgM, original magnification ×500 (b); IgD, original magnification ×500 (c).

The peripheral blood demonstrated rouleaux formation as well as circulating plasma cells, some with cytoplasmic immunoglobulin inclusions (Russell bodies). The marrow aspirate smears contained numerous plasma cells with numerous Dutcher bodies and Russell bodies, as well as lymphoplasmacytic morphology (Figure 2(a)). The bone marrow biopsy demonstrated hypercellular marrow for the patient's age that was extensively replaced by an infiltrate of predominantly mature-appearing plasma cells, some of which were binucleated and containing Dutcher bodies, that accounted for 95% of the total cellularity on the core biopsy. Flow cytometry confirmed the presence of a population CD138 positive, CD38 positive, CD20 partially positive, CD19 negative, CD56 negative, and cytoplasmic kappa light chain restricted plasma cells with no abnormal B-lymphoid population identified. Immunohistochemistry performed on paraffin sections of the core biopsy demonstrated the neoplastic plasma cells to be diffusely positive for IgM (Figure 2(b)) and a subset that was weakly positive for IgD (Figure 2(c)). Interestingly, the areas with increased IgD positive cells were also CD20 positive. Cyclin D1 was positive on many of the plasma cells but highlighted fewer plasma cells as compared to IgM. Conventional G-band karyotyping of the bone marrow revealed a normal male karyotype, 46 XY. Using Vysis DNA probes, FISH analysis was positive for IGH break apart and CCND1/IGH fusion gene rearrangements in approximately 80% of cells examined, indicating a t(11;14). The presence of an extra 3′ signal for IGH corresponded to the finding of an extra fusion signal found in a proportion of cells examined for the CCND1/IGH gene rearrangement (46.1%), further supporting the presence of a t(11;14). FISH analysis was also positive for the loss of D13S319 along with loss of the control probe at 13q34, suggesting either a large deletion of the long arm of chromosome 13 or, alternatively, monosomy 13. FISH analysis was negative for loss of the TP53 tumor suppressor gene as well as for hyperdiploidy assessed by DNA probes specific for chromosomes 5, 7, and 9. At this point, the patient was felt to have a symptomatic plasma cell myeloma. Immediately, subsequent serum and urine protein electrophoresis with immunofixation was negative for IgD heavy chains. 

TPE, in conjunction with 6 cycles of cyclophosphamide, bortezomib, and dexamethasone chemotherapy, resulted in a decrease in his IgM and serum viscosity to 1159 mg/dL and 2.9 cP, respectively, with concomitant improvement in his mental status. Unfortunately, the patient did not achieve a durable response with these treatments, and by the seventh cycle of chemotherapy he had an exacerbation of his disease (IgM 5120 mg/dL and serum viscosity 7.3 cP). He received 3 additional TPE procedures and was subsequently switched to treatment with lenalidomide, but this was discontinued after only three days of therapy due to development of a skin rash. Bendamustine therapy was then initiated, but once again no clinical response was observed. Currently, the patient is undergoing a trial of thalidomide.

## 3. Discussion

The rarity of IgD/IgM biclonal PCM may be attributed to the fact that IgD and IgM paraproteins individually are among the rarest variants identified even in monoclonal plasma cell myeloma, representing 0.5% and 2% of cases, respectively [2]. Furthermore, in previous studies, biclonality was detected using serum or urine protein electrophoresis without direct immunohistochemical visualization of clonal populations [2, 5–8]. In the cases presented in this report, routine immunofixation electrophoresis studies only detected the IgM paraprotein. As occurred in Case  1, many laboratories, as a cost-saving measure due to the rarity of its secretion, do not routinely perform immunofixation for IgD once another paraprotein is detected, possibly leading to underdetection of an IgD paraprotein. If immunofixation for IgD is performed, as in Case  2, the lack of detectable IgD secretion by serum protein electrophoresis may be attributable to the decreased number of IgD positive neoplastic cells when compared to the IgM positive cells or may represent a minimally secretory or nonsecretory clone [1]. 

These IgD/IgM biclonal cases shared some characteristic morphologic and immunophenotypic features which both raised and resolved a differential diagnostic consideration. Both cases displayed lymphoplasmacytic morphology and prominent Dutcher and Russell bodies. The lymphoplasmacytic morphology in these cases leads to a potential diagnostic dilemma. The phenomenon of biclonal gammopathy is not restricted to plasma cell neoplasms, as other B-cell lymphoma with plasmacytic differentiation can produce paraproteins, which on occasion can result in biclonal gammopathy [10]. Lymphoplasmacytic lymphoma, the prototypic lymphoma with plasmacytic differentiation, has been reported to present with biclonal gammopathy, including IgD with IgM [11, 12]. In the light of the overlapping morphologic features and serum monoclonal paraproteins, it may be difficult to separate IgD/IgM plasma cell myeloma from a B-cell lymphoma with plasmacytic differentiation; however, this distinction is extremely important given the differences in how these two entities are currently treated. Fortunately, in addition to an immunoprofile characteristic of plasma cell myeloma (diffuse positivity for CD138, negativity of CD19 and dim to negative expression for CD45), both cases demonstrated cyclin D1 expression, which, while characteristic of mantle cell lymphoma and hairy cell leukemia, is not a feature of B-cell lymphoma that commonly shows plasmacytic differentiation, such as lymphoplasmacytic lymphoma [10]. Cyclin D1 expression is found in a subset of plasma cell myelomas, especially those with lymphoplasmacytic morphology [13]. Although the finding of cyclin D1 expression in both cases is interesting, it was only related to a t(11;14) in Case  2, suggesting other mechanisms besides the rearrangement of the *CCND1* gene for its overexpression. This observation is in line with prior studies which show a high incidence of t(11;14) reported in IgE, IgM, and non-secretory plasma cell myelomas, but not IgD myeloma [14]. 

In summary, these two cases illustrate that PCM may rarely exhibit biclonal expression of IgD and IgM heavy chains; a fact of which hematologists and hematopathologists should be aware. As the expression of IgD and IgM is more characteristic of B-cell lymphoma, particularly those that may be associated with Waldenström's macroglobulinemia such as lymphoplasmacytic lymphoma, a diagnostic dilemma exists. Paraffin section immunohistochemistry is an essential ancillary study, as serum protein electrophoresis may miss cases with biclonality. Clinically, both of our cases were chemotherapy-resistant, suggestive of more aggressive clinical courses; however, it is difficult to characterize the clinical behavior of IgD/IgM PCM based on this small sample size. With the increased awareness of this entity, additional data will expectedly emerge and provide better characterization of the clinicopathologic features and behavior of this rare disease.
